# Performance effects of simulation training for medical students – a systematic review

**DOI:** 10.3205/zma001572

**Published:** 2022-11-15

**Authors:** Niall McInerney, D. Nally, M.F. Khan, H. Heneghan, R.A. Cahill

**Affiliations:** 1 Mater Misericordiae University Hospital, UCD Centre for Precision Surgery, Dublin, Ireland; 2 Mater Misericordiae University Hospital, Department of Surgery, Dublin, Ireland; 3 University College Dublin, School of Medicine, Section of Surgery and Surgical Specialties, Dublin, Ireland; 4 St. Vincent’s University Hospital, Department of Surgery, Dublin, Ireland

**Keywords:** undergraduate, medical education, simulation, performance

## Abstract

**Objective:**

Simulation based medical education (SBME) is fast becoming embedded into undergraduate medical curricula with many publications now describing its various modes and student self-reported impacts. This systematic review synthesizes the available literature for evidence of performance effects of SBME as an adjunct within traditional teaching programmes.

**Methods:**

A narrative systematic review was conducted according to PRISMA guidelines using Ovid MEDLINE, EMBASE, and PubMed databases for studies, published in English, reporting on general medical and surgical undergraduate SBME between 2010 to 2020. Two reviewers independently assessed potential studies for inclusion. Methods and topics of simulation with their assessments were evaluated. Descriptive statistics were used to describe pooled student cohorts.

**Results:**

3074 articles were initially identified using the search criteria with 92 full-text articles then screened for eligibility. Nineteen articles, including nine randomised trials, concerning 2459 students (median 79/study), were selected for review. Cardiac scenarios were commonest (n=6) with three studies including surgical topics. Nine studies used mannequin simulators (median time/session 17.5minutes) versus standardised patients in seven (median time/session=82 minutes). Educational impact was measured by written (n=10), checklist (n=5) and OSCEs (n=3) assessment either alone or in combination (n=1, OSCE/written assessment). All articles reported a positive effect of SBME on knowledge including improved retention in three.

**Conclusion:**

SBME, as an adjunct to existing curricula, improves knowledge-based performance of medical students at least in the short-term. Future studies should broaden its topics, assess longer term impacts and cost-effectiveness while also considering whether and what areas of traditional undergraduate learning it can replace.

## Zusammenfassung

**Zielsetzung:**

Die simulationsbasierte medizinische Ausbildung (SBME, Simulation Based Medical Education) wird zunehmend zu einem festen Bestandteil des vorklinischen Curriculums. Es gibt inzwischen viele Veröffentlichungen, in denen die verschiedenen Formen der simulationsbasierten medizinischen Ausbildung und die von den Studierenden selbst beschriebenen Auswirkungen beschrieben werden. In dieser systematischen Übersichtsarbeit wird die verfügbare Literatur hinsichtlich nachweislicher Leistungsauswirkungen der SBME zusammengefasst, die als Ergänzung zu traditionellen Lehrprogrammen eingesetzt wird.

**Methodik:**

Es wurde eine narrative systematische Übersichtsarbeit gemäß den PRISMA-Leitlinien durchgeführt, bei der die Datenbanken Ovid MEDLINE, EMBASE und PubMed nach englischsprachigen Studien durchsucht wurden, die zwischen 2010 und 2020 über allgemeinmedizinische und chirurgische SBME-Maßnahmen in der Vorklinik berichtet haben. Zwei Reviewer werteten unabhängig voneinander potenzielle Studien für die Aufnahme in die Übersichtsarbeit aus. Es wurden Methoden und Themen der Simulation mit ihren Bewertungen untersucht. Zur Beschreibung gepoolter Studierendenkohorten wurde deskriptive Statistik verwendet.

**Ergebnisse:**

Anhand der Suchkriterien wurden zunächst 3074 Artikel ermittelt, von denen 92 Volltextartikel auf ihre Eignung geprüft wurden. Neunzehn Artikel, darunter neun randomisierte Studien, an denen 2459 Studierende teilnahmen (Median 79 pro Studie), wurden zur Auswertung ausgewählt. Am häufigsten waren kardiologische Szenarien (n=6). Drei Studien umfassten chirurgische Themen. In neun Studien wurden Simulationspuppen verwendet (mittlerer Zeitaufwand pro Sitzung: 17,5 Minuten), während in sieben Studien standardisierte Patienten eingesetzt wurden (mittlerer Zeitaufwand pro Sitzung: 82 Minuten). Die ausbildungsrelevanten Auswirkungen wurden durch schriftliche Bewertungen (n=10), Checklisten (n=5) und OSCE-Bewertungen (n=3) einzeln oder in Kombination (n=1, OSCE-Bewertung/schriftliche Bewertung) gemessen. Alle Artikel berichteten über eine positive Auswirkung der SBME auf die Wissensentwicklung. In drei Artikeln wurde darüber hinaus über eine verbesserte Beibehaltung von Wissen berichtet.

**Schlussfolgerung:**

SBME als Ergänzung zu bestehenden Curricula verbessert zumindest kurzfristig die wissensbasierte Leistung von Medizinstudierenden. Künftige Studien sollten die diesbezüglichen Untersuchungsthemen ausweiten, die längerfristigen Auswirkungen und die Kosteneffizienz bewerten und zudem auch prüfen, welche Bereiche der vorklinischen Ausbildung die SBME gegebenenfalls ersetzen kann.

## Introduction

Medical education largely still follows traditional structures [1], [2]. Students undergo didactic lecture-based learning throughout their studies but especially in their early years [3]. Once on clinical sites, they learn by engaging with clinical teams and real patients. Although proven sufficient over time, the acquisition of medical knowledge and skills in this way has a number of potential pitfalls. Students are expected to learn from and practice recently acquired knowledge and skills on actual patients. This interaction may be complicated both ways– medical students can be nervous and patients, a vulnerable cohort, can be fearful. Furthermore, the clinical experience may be variable between teams and over time and much of the interaction happens without direct observation by academic faculty. The heterogeneity and inconsistency of clinical exposure coupled with lack of assessment-relevant feedback before examinations is suboptimal and may undermine fairness in competitive assessments and standards in future medical practice. Also, the COVID-19 pandemic has greatly challenged medical undergraduate programmes and students by the withdrawal of ward-based placements.

To improve their skills in both history taking and physical examinations, students have long practiced individually and with their peers. This provides a safe, comfortable environment for them to hone their skills and enables iterative improvement by doing, although this again lacks senior supervision and standardisation and may not challenge. Simulation has long been used in aviation and military training and is fast becoming a formal component of undergraduate and postgraduate medical education [4], [5], [6]. Simulation training providing a “device that presents a simulated patient (or part of a patient) that interacts appropriately with the actions taken by the simulation participant” allows users to learn in a safe, controlled and standardised environment, so that skills and knowledge can be applied and practiced [7]. Recent technological advances have increased the capability to realistically mimic actual patients and real-life clinical scenarios [8], [9]. 

While postgraduate simulation has been studied extensively in the literature (with some of its proven benefits including greater patient safety, improved teamwork and enhanced confidence [10], [11], [12], [13], [14], [15]), there is less evidence detailing the effect of such training on undergraduate medical student performance. Although many previous studies have detailed the self-reported effects of simulation training on medical students [10], [16], [17], objectively assessed impacts need to be established prior to its broad implementation most particularly to justify the necessary expenditure but also especially if it’s to replace other existing curricular components, either by design or necessity (eg due to public health emergencies). Crucial to any education innovation is assurance of benefit. The primary metric of performance in medical education are assessment scores. The purpose of this review is to synthesise the available evidence on the performance effect of simulation based medical education (SBME) as applied to technical, procedural and examination skills for undergraduate medical students in general medicine and surgery. 

## Methods

### Search database

This systematic review was conducted using the Preferred Reporting Items for Systematic Review & Meta-Analyses (PRISMA) guidelines. The searches were performed independently in duplicate (NM, FK) using OVID, EMBASE and PubMed databases from 2010 to 2020 inclusive. The final search was completed in January 2021. All eligible records were screened independently (NM, FK) for relevance.

#### Search terms

The following Medical Subject Headings terms and keywords were used: medical education OR medical students OR medical student AND simulation training OR high fidelity simulation training OR mannequin OR manikins OR SimMan or simulation. Boolean AND/OR operators were used to combine MeSH and terms and keywords. Following the search, titles and abstracts were screened. Full text of potentially eligible articles were reviewed by two authors (NM, FK) independently and eligible studies selected.

#### Inclusion/exclusion criteria

Our inclusion criteria required all articles to be in English, to be research-based and to include objective investigation of the efficacy of a simulation-based training programme for undergraduate medical students in general medical and surgical clinical learning stages regarding clinical performance. Studies which assessed history taking, physical examination, and clinical practice either separately or together were included regardless of method of simulation (including whether mannequins or simulated patients were used) and assessment (i.e. whether written, Objective Structured Clinical Examination (OSCE) or tutor assessment). OSCEs in the included students followed the traditional format described by Harden [18].Studies assessing procedural protocols (including BLS, ACLS and ATLS) and surgical skills were excluded. Checklist assessments were included but studies applying exclusive subjective assessment methodology (eg overall global rating) were excluded. Studies assessing subspecialty domains such as obstetric, paediatric, anaesthetic and psychiatric simulation were excluded. 

#### Data collection 

The following data was extracted from each included publication: first author, publication year, country, study design, number and stage of students, simulation method, assessment method, performance levels pre and post simulation, effects on knowledge retention, students’ confidence and authors’ conclusion. 

## Results

### Study characteristics

**Figure 1:**
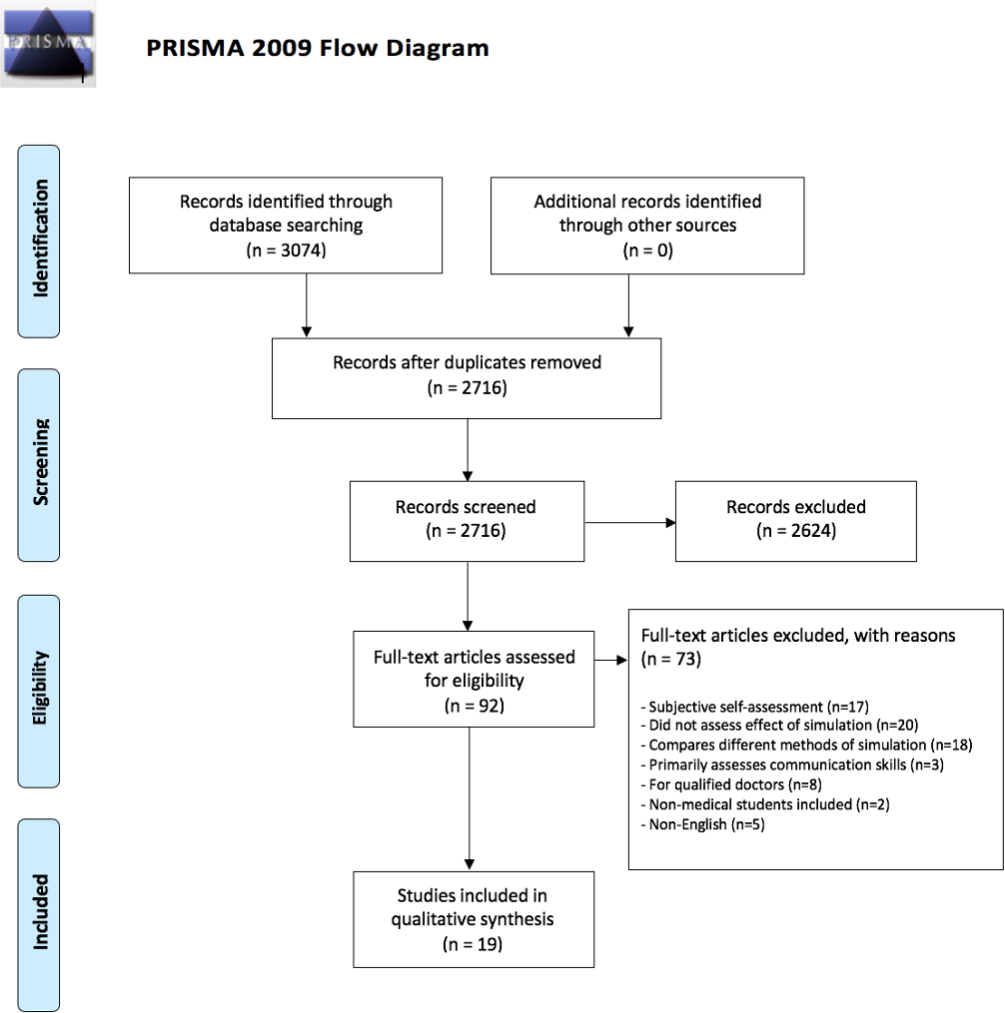
PRISMA flowchart of the search and selection process [50]

Figure 1 (Fig. 1) shows the PRISMA flowchart of search and selection process. 3074 studies were identified through database searching. Following removal of duplicates, the abstracts of 2716 studies were evaluated and included if deemed suitable. The full text of 92 articles were assessed for eligibility, and ultimately 19 articles were included for qualitative synthesis. 

Table 1 (Tab. 1) summarizes the final nineteen included studies comprising 2459 students ranging from first to final year medical students. Studies from eleven countries were included with three focusing on first (n=2) or second year (n=1) students and the remainder concerning those in later years. There were nine randomised control trials [19], [20], [21], [22], [23], [24], [25], [26], [27], six prospective cohort studies [28], [29], [30], [31], [32], [33], two crossover studies [34], [35], one retrospective analysis [36], and one case-control study [37]. The median number of patients per study was 79 (20-615 patients). Due to heterogeneity of study type including the variety of tools used for assessing multiple skills, meta-analysis was prohibited. Attachment 1 summarizes the results from each study. 

#### Specialities

Medical scenarios were the most common topics for simulation with fourteen groups [28], [36], [30], [31], [22], [32], [24], [34], [33], [25], [26], [35], [27], [37], conducting simulation training based around common medical pathologies. Of these, six groups simulated cardiac scenarios with four of these assessing auscultation skills [20], [30], [34], [27] and two simulating acute cardiac presentation scenarios [32], [26]. Surgical topics were simulated in two groups [19], [29] while one group [23] used both medical and surgical scenarios for their simulation sessions.

#### Methods of simulation

Various different methods of patient simulation were used with the majority (n=12) using artificial patient models in scenarios to mimic the medical setting lasting a median of 17.5 minutes (range 15-30 mins) [28], [21], [36], [30], [31], [22], [24], [34], [33], [35], [27], [37]. SimManTM (Laerdal) was the most commonly used simulator (n=6) [36], [30], [24], [34], [35], [27]. Harvey (n=2) [33], [37], METI (n=1) [22] and Kyota kagaku (n=1) along with a heart-sound simulator (n=1) were the other artifical simulators used. SimMan is a wireless, life-sized advanced patient simulator, that can display physiological changes that the “patient” undergoes in real-time on a monitor, under the control of the simulation facilitator [https://laerdal.com/us/products/simulation-training/emergency-care-trauma/simman/]. In all six groups who used SimMan, general medical scenarios involved students taking a history and performing a physical examination [36], [30], [24], [34], [35], [27]. In these, the SimMan displayed abnormal cardiovascular and respiratory signs based on the simulated scenario. In two studies [30], [27], students were given a short orientation (15-30 minute) to clinical practice using a SimMan.

Standardized patients were used in seven studies [19], [20], [29], [23], [32], [25], [26], three of which [20], [29], [23] focussed on assisting clinical examination practice. Standardized patients methods ranged from actors [19], [36], [26] or academic staff mimicking learned symptoms to expert patients and focused on standardised patient histories [25] and ward rounds [19] with supplementary material such as drugs charts, patients’ vital parameters and end of bed notes being made available to students in all studies. The median time for simulation with standardized patients was 82.5 minutes (15-180 minutes). Giblett et al. [24] cumulatively spent 21 hours over the course of one semester simulating encounters with standardized patients. Giblett et al. [29] and Nassif [20] used standardized patients in conjunction with breast models to educate students on breast examination. 

#### Methods of assessment

Various methods of assessments were used to evaluate the performance effect of simulation training. Written assessment was the most common (n=10) [28], [29], [31], [22], [32], [24], [34], [33], [25], [26], [35], predominantly comprising of an MCQ examination. Four groups [19], [23], [25], [36] used a checklist assessment, which was either completed during or after the simulation scenario. Three groups [20], [23], [37] assessed their students using OSCE examinations alone. One group [21] used a combination of OSCE and written assessment. 

#### Effect on performance

All groups reported a performance benefit to students associated with simulation training. Simulation training was shown to have a positive effect when used across a broad range of medical and surgical specialities, in acute and non-acute scenarios. 

#### Auscultation simulation

Swamy [35] reported improved results on a knowledge-based questionnaire following clinical chest examination training with SimManTM compared to examining their student colleagues, later confirming these results in a further larger cohort [24]. There was also improvement noted in the simulation group’s self-perceived confidence. In a cross-over trial, at the mid-test point, the group who performed examinations on mannequin performed significantly higher on a knowledge assessment then those who performed peer examinations. Perlini [32] also demonstrated the impact simulation training has on retention of knowledge, focusing on cardiac auscultation. After three years, a subgroup of his students were reassessed. Without any further exposure to the Harvey simulation over that timeframe, retention of the acquired capability was maintained. Pereira [31] also showed a positive effect of simulation on cardiac auscultation. When comparing pre and post test scores, there was a 16% performance improvement when simulation training is added to the existing curriculum. Equally, Bernardi [28] demonstrated an improvement in cardiac auscultation skills when practiced on a simulator. There was however no improvement in respiratory auscultation between the simulation and control group. Kern et al. [37] implemented a cardiac auscultation programme following previous reports of deficiencies in physician’s clinical examination skills [38], [39]. In this, students who received simulation training (using the Harvey simulator) along with the standard curriculum were compared to students who received the standard curriculum alone. To ensure little variation in teaching between the groups, the same three faculty teachers facilitated teaching in the same facility for all students. Students were assessed in a multi-station OSCE five weeks following their respective learning. Students who received simulation training performed significantly better in the respective assessed cardiac skills than the control group. Again, there was no difference in pulmonary examination skills. 

#### Breast examination simulation

Nassif [20] used a hybrid simulation model of breast examination where a standardized patient wearing a silicone breast simulator jacket was examined. This group was compared to students who examined a standardized tabletop breast model. Following this intervention, both groups were assessed in an OSCE. Students who participated in hybrid simulation training were significantly better at lesion reporting, identification of malignant features and accurate location identification compared to the group who received traditional teaching. Angarita [21] also evaluated the effect of simulation training on students’ clinical breast examinations. Students were taught using a simulation and multimedia-based curriculum, which was compared to the traditional didactic lecture and clinic-based teaching. Both groups were assessed using written and OSCE assessments. The group who completed the simulation-based training were significantly better at all aspects of the breast exam (including inspection, position, palpation, pressure, axillary exam and providing justifications for performing a breast exam). Additionally students who underwent simulation training were significantly more confident than their peers who were taught with traditional methods. Alluri [22] used simulation to teach pre-clinical medical students, and assessed the effect in a randomised, controlled cross-over study finding that both simulation and didactic lectures improved student knowledge when assessed on an MCQ. When assessing delayed test scores, thus evaluating retention of knowledge, students who completed simulation training demonstrated improvement, those who were taught didactically did not. 

#### Simulation of emergency scenarios

Vattanavanit [36] assessed sixth year medical students knowledge and confidence in septic shock resuscitation. Students who received simulation training improved significantly in knowledge and resuscitation skills, whilst also improving their confidence at assessing patients in septic shock (Post-simulation 68.1%±12.2% vs pre-simulation 5.64±13.1, p<0.001). Solymos [24] looked at the area of critical medicine, comparing simulation-based teaching to traditional didactic teaching. Final year students were evaluated using a multiple-choice questionnaire – at baseline, post-teaching and a two week follow up. Although there was a significant improvement following simulation compared to the didactic lecture group, baseline scores were higher in the didactic lecture group. McCoy [27] performed a cross-over study, particularly focusing on assessing critically unwell patients with myocardial infarction or anaphylaxis. Simulation training was compared to traditional didactic lectures. Students’ performance was evaluated in real-time during the simulation. 96% of students performed better when trained with simulation. Overall, simulation training resulted in a 22% absolute increase in scores (95% CI 18-26%). History taking (27% absolute increase in score), physical examinations (26%) and patient management (16%) components of the assessment were higher in the simulation group compared to the lecture group. DeWaay [26] investigated fourth year medical student performance in students who received simulation training compared to a control group (who received no intervention) and to a group who received didactic lectures. Simulation significantly improved overall performance. The percentage of correct answers in the simulation group was 53.5±8.9% compared to 47.9±9% in the didactic teaching group and 47.9±9.8% in the control group (p<0.001). Williams [32] also simulated cardiac emergencies, but this time using real patients with a cardiac history taking the simulated patient role. Students were assessed using knowledge based short answer questions. Mean scores increased (25/43 to 34/43) after the intervention. A sustained effect was seen at one week post intervention, with scores of 35/43. Students self-perceived confidence was also improved post intervention. Sanchez-Ledesma [30] focused on the use of simulation training in the management of neurological emergencies. The simulation instructor evaluated students’ during the simulation session. Once again, statistically significant difference were found between pre and post-test groups with results improving further following repeated simulation sessions.

#### Simulation in non-emergent scenarios

Simulation training was not limited to acute medical presentations. Fisher [25] developed and delivered a simulation programme dealing with common geriatric issues, including delirium, falls and elder abuse. Mannequins and simulated patients were both incorporated into the scenarios. Students were assessed pre, post and one month post-simulation. Test scores were compared to those who underwent traditional didactic teaching with post simulation test scores being better than pre-simulation test scores. For all scenarios, there was a statistically significant difference between the simulation and control group (p<0.005). Students in Zhang et al. [23] study were pre-selected into a simulation and didactic lecture group by virtue of variants in facility across their clinical sites. Students simulated both medical and surgical scenarios. Across two year groups, the mean score for 16 OSCE stations was significantly better in those who had undergone simulation training. The mean score in 2013 for the simulation was 80.95±0.61 versus 69.91±1.24 for the didactic lecture group (p=0.0114), and 86.12±0.56 versus 73.58±1.34 in 2014 (p=0.006). 

#### Simulation in surgical education

Two studies specifically examined simulation in surgery. Giblett [29] randomised two groups of medical students in their first year of clinical attachments. In the first semester one group received traditional didactic lecture-based education while the other group received simulation training, broadly covering the surgical curriculum. Using independent t-test analysis, a significant performance benefit in a knowledge-based assessment was seen amongst the group who received simulation training (p<0.001). Additionally, the simulation group had higher self-reported confidence and understanding of surgical principles. These students also showed substantially improved confidence in acute surgical assessments, particularly in abdominal (p<0.001), vascular (p<0.001) and breast examinations (p<0.001). Grunewald [19] used an objective surgical ward round assessment tool to evaluate students’ performance. The control group did not receive simulation training. Competence in the intervention group improved from 62.6 to 69.6 points (p=0.0169). In contrast, there was no improvement in the control group (pre: 62.6 vs post 69.6 points (p=0.72)). 

## Discussion

SBME is of increasing interest for medical undergraduate programmes. This has been especially the case recently with the COVID-19 pandemic pressurising clinical placements with added emphasis on graduating competent doctors in a timely fashion and indeed even early. The primary outcome of this study was to examine for evidence of performance effect of simulation training on medical student performance through a synthesis of the published literature including summarising the methods used to provide simulation training and the tools used to assess efficacy. As evidenced by this review, simulation training in tandem with the traditional curriculum has been shown to generally improve medical students' performance and knowledge retention alongside confidence over didactic teaching and learning through observation. These benefits can be seen across a number of required skills including core components such as history taking and physical examination (including essential, intimate physical examinations, such as a breast examination which can be otherwise challenging for student to learn) and across various specialities in both emergency and elective general medical and surgical situations. Furthermore, students who experienced simulation training have been found to be more satisfied with their teaching [29]. These findings shouldn’t perhaps be surprising as students learn best when they are actively involved [40]. While medical training has traditionally utilised the adage “see one, do one, teach one”, simulation-based education provides the opportunity to “do one” repeatedly, safely and under supervision to improve future practice. 

SBME of course requires some investment in terms of teaching personnel, equipment and space meaning objective proof of its usefulness is very important to justify the expenditure. Additionally nuance exists. Hamstra [41] detailed some of the key components to effectively run simulation scenarios. Learner engagement and a suspension of disbelief enhance the learning environment for medical students. By placing them in scenarios and an environment that mimics real life, a superior educational experience can be obtained. Also some studies have suggested that improved student confidence may be a negative finding [42] indicating that further work needs to be done in this specific area. Furthermore, while simulation training as been shown to improve cardiac auscultation skills, it seems to have no effect on respiratory auscultation skills [28], [31], [33], [37]. Bernardi [28] hypothesised that this difference is related to the different teaching methods employed for each and that using graphic representation of the lung sounds heard may offset this. Further, additional “real-world” validity can be added when constructing the scenarios (for example, Williams simulated cardiac emergencies with real patients who had recovered following a previous emergency cardiac presentation including intermittent interruption of the students to simulate a real life “on call scenario” as doctors are often required to multi-task, manage their time efficiently and remain calm under pressure [23]).

As much as simulation training facilitates the standardization of medical education, in allowing all students access similar clinical experiences, it would seem also to provide a useful means of contributing to student summative assessment in a manner that is reproducible and objective. To date, written examinations in conjunction with observed clinical examination and skills assessment have traditionally been major components of medical students' assessment [43], [44]. Recently, two studies have indicated that a simulation-based assessment may be appropriate for assessing clinical competence [45], [46]. In addition, the healthcare educator’s primary function is to produce competent and proficient doctors and the physical and mental wellbeing of our students is increasingly recognised as essential given the increasing rates of burnout and mental health issues being reported amongst medical students [47], [48]. A consensus statement on medical student wellbeing from the Australia and New Zealand [49] recommends “curricula that promote peer support and progressive levels of challenge to students and to employ strategies to promote positive outcomes from stress and to help others in need”. These strategies are already components of SBME and further aspects such as resilience training can be readily incorporated. Another area to examine further relates to whether confidence improvement by simulation can help ease the transition from medical student to junior doctor. 

In conclusion, this systematic review provides evidence that SBME can improve medical students’ performance in a variety of domains and specialities while also identifying areas in need of future address. Alongside performance benefits in history taking and physical examination, there is evidence to show that SBME leads to greater knowledge retention and confidence. Therefore this review validates the use of SBME as an adjunct to the traditional didactic lecture-based curriculum. For the purposes of ensuring optimal education of medical students, further studies could investigate the best methodologies for SBME by comparison and whether simulation is best employed as an adjunct or replacement to the traditional lecture-based curriculum. It is important also to examine cost-effectiveness especially the role of lower cost set-ups versus more expensive systems. Ultimately too it is important to correlate the performance effect of simulation training directly to competence. By building up such an evidence-base we will best evolve the curriculum for the purpose of producing better doctors, and most importantly, better patient outcomes.

## Limitations

This systematic review studies heterogenous groups consisting of various methods of simulation and assessment. As such meta-analysis was prohibited. 

## Acknowledgements

The authors would like to thank Angela Rice, Library and Information Services, Mater Misericordiae University Hospital for her guidance during this project. 

## Competing interests

The authors declare that they have no competing interests. 

Professor Ronan Cahill is named on a patent filed in relation to processes for visual determination of tissue biology, receives speaker fees from Stryker Corp and Ethicon/J&J, research funding from Intuitive Corp and Medtronic and holds research funding from the Irish Government (DTIF) in collaboration with IBM Research in Ireland and from EU Horizon 2020 in collaboration with Palliare.

## Supplementary Material

Tabulated summary of intervention with method of assessment along with outcome from each included study

## Figures and Tables

**Table 1:**
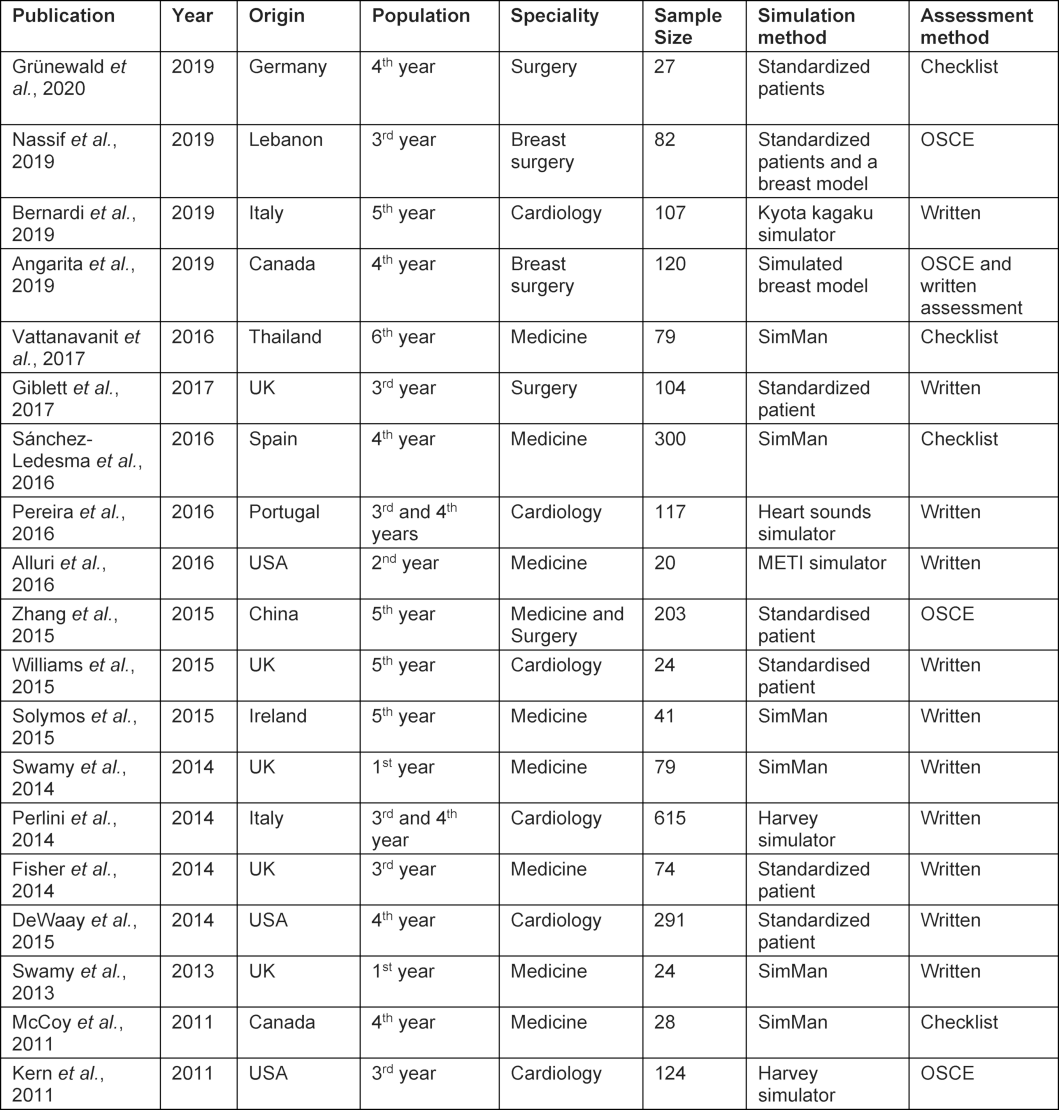
Table summary of included publication study characteristics
