# Supplementary material for: Performance effects of simulation training for medical students – a systematic review
Source: GMS J Med Educ. 2022 Nov 15;39(5):Doc51. doi: 10.3205/zma001572 (PMC9733478; doi:10.3205/zma001572)
Supplement: Tabulated summary of intervention with method of assessment along with outcome from each included study [file JME-39-51-s-001.pdf]

Attachment 1: Tabulated summary of intervention with method of assessment along with outcome from each included study

Author	Assessment method	Non-simulation group scores	Simulation group scores	Effect on performance	Self-perceived confidence	Knowledge retention
Grunewald (Grünewald *et al.*, 2020)	Checklist	n=11<br>Difference in pre and post intervention scores<br>-1.2 +/-11.1	n=16<br>Difference in pre and post intervention scores<br>6.9+/-10.3	Difference of 8 points on SWAT assessment between groups<br>p = 0.061	Confidence at conducting surgical ward round following intervention:<br>Control group: 0.7+/-0.4 (p 0.088)<br>Intervention group: 4.8+/- 0.4 (p<0.001)	
Nassif (Nassif *et al.*, 2019)	OSCE	n=26<br>CBE completeness score: 16.83<br>Visual inspection score: 4.83<br>Palpation score: 11.67<br>Lesion identification grade: 1<br>Reporting a lesion: 0.5<br>Malignant features of the lesion: 0.00<br>Accurate location of the lesion: 0.33<br>Falsely reporting a lesion: 0.00<br>Detecting lymph nodes: 1.0<br>Falsely reporting lymph nodes: 0.00	n=56<br>CBE completeness score: 16.58<br>Visual inspection score: 5<br>Palpation score: 11.5<br>Lesion identification grade: 3<br>Reporting a lesion: 1<br>Malignant features of the lesion: 0.67<br>Accurate location of the lesion: 1.33<br>Falsely reporting a lesion: 0.00<br>Detecting lymph nodes: 1.0<br>Falsely reporting lymph nodes: 0.00	Simulation group were significantly better at lesion identification, identification of malignant features, and accurate location identification (p<0.001	11.11% of simulation group feel more prepared for their upcoming examination compared to 3.85% of the control group (p=0.418)	
Bernardi (Bernardi *et al.*, 2019)	Written	Cardiac auscultation<br>Aortic stenosis: 77.6%<br>Mitral regurgitation: 71.4%<br>Split heart sounds: 79.6%<br>Respiratory auscultation<br>Wheeze: 91.7%<br>Fine crackles: 58.3%	Cardiac auscultation<br>Aortic stenosis: 84.5%<br>Mitral regurgitation: 89.7%<br>Split heart sounds: 89.7%<br>Respiratory auscultation<br>Wheeze: 91.4%<br>Fine crackles: 63%	Training with a patient simulator improved students' cardiac auscultation skills, in particular auscultating for aortic stenosis (p=0.36), mitral regurgitation (p=0.02) and split heart sounds (p=0.15)		Performances of the same experimental students between year three and year five, there were no changes in the heart auscultation results, whereas they significantly improved over time in lung auscultation.

		Coarse crackles: 66.7%	Coarse crackles: 70.7%	Training with a patient simulator did not significantly improve students' lung auscultation skills.		
Angarita (Angarita *et al.*, 2019)	OSCE and written assessment	n=58<br>Percentage of students who adequately completed all 13 items: 28.2%	n=62<br>Percentage of students who adequately completed all 13 items: 88.2%	Students in simulation group adequately completed all 13 items was significantly higher than the control group (p<0.00001)	Students who underwent simulation training were "very confident" more frequently than traditionally trained students (97.1% vs 9.6%, p=0.00001)	
Vattanavanit (Vattanavanit, Kawla-led and Bhurayanontachai, 2017)	Checklist		Pre & post-test analysis<br>Mean test score significantly improved following simulation training<br>(66.83% +/-19.7% vs 47.59%+/-19.7%, p<0.001)		Student confidence in the management of septic shock significantly better following simulation training (68.10%+/-12.2% vs 51.64+/-13.1%, p<0.001).	
Giblett (Giblett, Rathore and Carruthers, 2017)	Written	n=50<br>Median score=17 (43%)<br>Ranger 5-29 (13-74%)	n=39<br>Median score=29 (74%)<br>Range 16-35 (41-90%)	p<0.001	Confidence improvement in all domains of evaluated (including acute abdomen, assessing breast lumps, assessing peripheral vascular disease.<br>(p<0.001).	
Sanchez-Ledesma (Sánchez-Ledesma *et al.*, 2016)	Checklist		Pre-test scores<br>2014: 5<br>2015: 10<br>Post-test scores<br>2014: 12<br>2015: 28	Statistically significant difference found between pre-test and post-test groups (p<0.05)		

Pereira (Pereira *et al.*, 2016)	Written	Pre-test scores<br>Normal: 40.17%<br>Pathology unspecified: 14.02%<br>Mitral regurgitation: 7.18%<br>Aortic regurgitation: 5.47%<br>Aortic stenosis: 4.1%<br>Pulmonic stenosis: 6.32%<br>VSD: 5.47%	Post test scores<br>Normal: 56.92%<br>Pathology unspecified: 31.28%<br>Mitral regurgitation: 10.26%<br>Aortic regurgitation: 7.52%<br>Aortic stenosis: 4.96%<br>Pulmonic stenosis: 4.27%<br>VSD: 2.05%	Significant improvement (+16%) in the differentiation between normal and pathological cases.		
Alluri (Alluri *et al.*, 2016)	Written	n=20<br>Pre-intervention: 41.17+/-20.42%<br>Post-test: 66.67+/-19.49%<br>Delayed post-test: 67.71+/-22.33%	n=20<br>Pre-intervention: 40.00+/-21.89%<br>Post-test: 55.00+/-18.02%<br>Delayed post-test: 79.17+/-18.76%	Average individual student improvement from post-test to delayed post-test significantly improved in simulation group compared to lecture (p=0.036)		When assessing delayed test scores, thus evaluating retention of knowledge, students who completed simulation training demonstrated improvement, those who were taught didactically did not.
Zhang (Zhang *et al.*, 2015)	OSCE	2013: n=36<br>69.91+/-1.24<br>2014: n=27<br>73.58+/-1.34	2013: n=73<br>80.95 +/- 0.61<br>2014: n=67<br>86.12+/- 0.56	p = 0.0114<br>p = 0.006		
Williams (Williams *et al.*, 2015)	Written		n=24<br>Pre-simulation:25/43<br>Immediately post-simulation:34/43<br>>1 week post simulation:35/43<br>p<0.001	Improvement in all domains assessed, including management of MI, LV failure, fast AF, medical emergencies and starting as an FY1 doctor.<br>p<0.001		
Solymos (Solymos, O'Kelly and Walshe, 2015)	Written	Baseline MCQ score: 17+/-3<br>Post didactic lecture MCQ score:<br>21.5+/-3.1	Baseline MCQ score: 14.3+/-2.2<br>Post-simulation MCQ score: 21.1+/-1.8	Significant difference in the improvement from baseline and post teaching MCQ in the simulation group compared to lecture 6.8 (21.1-14.3) vs. 4.5 (21.5-17), p=0.0387		2-week follow up MCQ were lower in both groups than post teaching results. Smaller margin in the simulation group 1.3 (19.8-21.1) vs. 3.6 (17.9-21.5), but not statistically significant (p=0.167).

Swamy (Swamy *et al.*, 2014)	Written	Pre-test score: 6.5<br>Mid-test score: 7.2	Pre-test score: 6.7<br>Mid-test score: 9.3	The group who simulated examinations on a mannikin performed significantly better than the group who performed peer examinations in the mid-test.	Confidence in examining patients improved significantly in the simulation group compared to the control group.	
Perlini (Perlini *et al.*, 2014)	Written	Pre-simulation training percentage of correct diagnoses: 11%	Post-simulation training percentage of correct diagnoses:<br>72%	Improved capability of correctly recognising cardiac diagnoses<br>(p <0.001)		After three years without any further simulation training, retention remained high (68.4%, p<0.001)
Fisher<br>(Fisher and Walker, 2014)	Written	Elder abuse management: 20%<br>Falls management: 25%<br>Delirium management: 20%	Elder abuse management: 36%<br>Falls management: 40%<br>Delirium management:52%	p=0.002<br>p 0.001<br>p 0.001	>95% of students felt better equiped to deal with geriatric scenarios	
DeWaay<br>(DeWaay DJ, McEvoy MD, Kern DH, Alexander LA, 2015)	Written	n=80<br>Overall performance score: 47.9+/-9.8%	n=147<br>Overall performance score: 53.5 +/-8.9%	Overall performance was significantly better with simulation training versus the control group (p<0.001)		
Swamy (Swamy *et al.*, 2013)	Written	n=12<br>Mean mid-test score:<br>5.66	n=12<br>Mean mid-test score:<br>6.75	Mid-test knowledge scores increased significantly between both groups (p<0.001), and the group who performed examinations on SimMan^{TM} performed better than the control group<br>No significant difference in post-test scores after crossing over, which demonstrates equal knowledge improvement after performing examinations on SimMan^{TM}	Both groups felt more confident differentiating between normal and abnormal signs after practicing on SimMan^{TM}	

McCoy (McCoy *et al.*, 2011)	Checklist	Mean overall score: 71%	Mean overall score: 93%	Significant improvement in student performance for those trained with simulation compared with those trained with traditional didactic lecture (p<0.0001)		
Kern (Kern *et al.*, 2011)	OSCE	Evaluation of point of maximal impulse: 64%<br>Anterior auscultation of four cardiac areas: 71%<br>Left lateral cardiac auscultation: 41%<br>Inspection for lower extremity edema: 52%<br>Inspection for jugular venous distention: 41%<br>Respiratory<br>Chest expansion: 89%<br>Percussion: 89%<br>Auscultation:99%	Evaluation of point of maximal impulse: 74%<br>Anterior auscultation of four cardiac areas: 85%<br>Left lateral cardiac auscultation: 52%<br>Inspection for lower extremity edema: 71%<br>Inspection for jugular venous<br>Distention: 56%<br>Respiratory<br>Chest expansion: 83%<br>Percussion: 82%<br>Auscultation 97%	Students who trained using cardiopulmonary simulator (SimSPLE) performed significantly better than the controls in all five cardiac exam skills: (a) evaluation of point of maximal impulse (p=0.045),<br>(b) anterior auscultation of four cardiac areas (p=0.003),<br>(c) left lateral cardiac auscultation (p=0.037), and inspection for<br>(d) lower extremity edema (p≤0.001), and<br>(e) jugular venous distention (p=0.004).<br>No statistically significant difference in pulmonary examination skills between the groups.		
